# Establishment and Characterization of a Novel Ovarian Germline Cell Line with Oogonial Stem Cell-like Properties from the Japanese Eel (*Anguilla japonica*)

**DOI:** 10.3390/biology15212022

**Published:** 2026-11-06

**Authors:** Zhaowei Zhong, Yizhen Lu, Dingyuan Luo, Zebin Chen, Shengkai Shi, Yangjie Xie, Hongwei Liu, Jincan Chen, Yonghua Jiang

**Affiliations:** 1 State Key Laboratory of Mariculture Breeding, Fisheries College, Jimei University, Xiamen 361021, China; zzw1996@jmu.edu.cn (Z.Z.); 202412951005@jmu.edu.cn (Y.L.); 202411710017@jmu.edu.cn (D.L.); 202515951014@jmu.edu.cn (Z.C.); 202321061029@jmu.edu.cn (S.S.); yjxie@jmu.edu.cn (Y.X.); 200261000048@jmu.edu.cn (H.L.); 2 College of Ocean and Earth Sciences, Xiamen University, Xiamen 361005, China; 3 Key Laboratory of Healthy Mariculture for the East China Sea, Ministry of Agriculture and Rural Affairs, Fisheries College, Jimei University, Xiamen 361021, China

**Keywords:** *Anguilla japonica*, oogonial stem cell-like cell line, germ cells, transcriptome, signaling pathways, self-renewal

## Abstract

The Japanese eel (*Anguilla japonica*) is an economically important aquaculture species, and its reproductive development and artificial propagation have received considerable attention. However, the absence of a stable in vitro female germ cell culture system has hindered investigations into oogenesis, reproductive regulation, and germplasm conservation. In this study, cells isolated from immature Japanese eel ovaries were enriched through differential adhesion, Percoll density-gradient centrifugation, and single-cell cloning, followed by optimization of culture conditions. A stable oogonial stem cell-like cell line of *A. japonica*, designated AjOSCL, was established and characterized via morphological observation, proliferation assays, colony-forming assays, karyotyping, immunofluorescence staining, alkaline phosphatase detection, and transcriptomic sequencing. AjOSCL maintained stable growth and self-renewal capacity during continuous culture for over 35 passages in vitro, with a cumulative culture duration exceeding 12 months, and retained a predominant chromosome number of 2*n* = 38 (consistent with the chromosome number of *A. japonica*), suggesting chromosomal stability during long-term culture. AjOSCL exhibited strong alkaline phosphatase activity and expressed germline-related markers (e.g., Vasa and Nanos2), together with pluripotency-related genes or proteins such as Nanog and Oct4. Transcriptomic analysis further revealed that AjOSCL displayed a molecular profile compatible with a germline stem cell-like phenotype, with enrichment of pathways related to germ cell maintenance and oocyte development, including ECM–receptor interaction, TGF-β signaling, Wnt signaling, and oocyte meiosis. Collectively, these results indicate that AjOSCL may serve as a renewable in vitro model for studying oogonial maintenance, offering a foundation for future investigations into female germ cell biology and germplasm conservation in *A. japonica*.

## Simple Summary

The Japanese eel (*Anguilla japonica*) is an economically important aquaculture species; however, its artificial reproduction remains challenging, largely because the mechanisms governing female germ cell development are still not fully understood. Oogonial stem cells are a type of female germ cell that can self-renew and contribute to egg development. In this study, we established an oogonial stem cell-like cell line from the ovary of the Japanese eel. The established cells were maintained for numerous passages (i.e., successive cell transfers over many months), formed colonies from single cells, retained a stable chromosome number, and expressed genes associated with germline identity and stem cell properties. They also preserved their growth and morphology after cryopreservation (freezing and storage in liquid nitrogen) and recovery. Collectively, these findings suggest that the established oogonial stem cell-like cell line offers a renewable laboratory model for studying female germ cell biology and reproductive regulation in the Japanese eel. This cell line may also facilitate future research on germplasm conservation (the preservation of genetic resources), reproductive biotechnology, and the improvement of artificial breeding strategies for this species.

## 1. Introduction

The Japanese eel (*Anguilla japonica*) is an economically important aquaculture species across East Asia, including China, with a highly specialized catadromous life cycle involving freshwater growth and marine reproduction [1,2]. In nature, female eels undergo long-distance spawning migration before completing gonadal maturation, whereas under aquaculture conditions ovarian development commonly arrests at the pre-vitellogenic stage and does not proceed to final maturation spontaneously [3,4,5]. Although exogenous hormones, including salmon pituitary homogenate and human chorionic gonadotropin, can induce gonadal maturation and enable the production of fertilized eggs and larvae, unstable oocyte quality, low fertilization rates, and poor larval survival remain major constraints on large-scale artificial propagation [2,5]. These issues indicate that further breakthroughs in Japanese eel artificial propagation depend not only on optimizing induced maturation and larval-rearing techniques, but also on a deeper understanding of the fundamental biological mechanisms underlying female germ cell development, maintenance, and differentiation. Nevertheless, systematic knowledge of oogonial self-renewal, oocyte formation, and related molecular regulation in the Japanese eel remains limited [6], highlighting the need for stable in vitro models with defined female germline characteristics.

Fish germline stem cells (GSCs), including spermatogonial stem cells (SSCs) and oogonial stem cells (OSCs), sustain gametogenesis through self-renewal and differentiation into subsequent germ cells [7,8,9]. The identification of continuously proliferating oogonia in the adult ovary of Japanese medaka (*Oryzias latipes*) provided important evidence for persistent oogonial stem cell activity in teleosts [10,11]. Since then, the isolation, identification, and culture of fish OSCs have become important research directions in reproductive biology and aquaculture biotechnology [12,13]. Unlike the controversial existence of ovarian GSCs in adult mammals, many teleosts maintain proliferative oogonia and continuous oogenesis throughout adulthood [14,15], providing a biological basis for germplasm conservation, surrogate reproduction, and germline chimera production [16,17]. Considerable progress has been made in germ cell culture and transplantation in species such as Japanese medaka [18], zebrafish (*Danio rerio*) [19], Yellow River carp (*Cyprinus carpio haematopterus*) [20], and Chinese tapertail anchovy (*Coilia nasus*) [21]. However, compared with SSCs, long-term culture of fish OSCs remains challenging. Ovarian tissues contain abundant granulosa, theca, and other somatic cells that complicate germ cell isolation and enrichment [12], while OSCs outside their native niche readily undergo differentiation, proliferative decline, or apoptosis [22,23]. Although ovarian germ cells have been isolated and cultured from species such as orange-spotted grouper (*Epinephelus coioides*) [24] and black rockfish (*Sebastes schlegelii*) [25], long-term ovarian cell models retaining clear oogonial stem cell-like characteristics remain scarce [12]. Therefore, establishing a stable in vitro culture system for fish OSCs remains one of the key issues to be addressed in this field.

Stable germ cell cultures provide reproducible platforms for studying cell proliferation, differentiation, fate regulation, and reproductive biotechnology. In Japanese eel, cell cultures have been established from several tissues, including the kidney and spleen [26], testis and ovary [27], but available models are largely somatic-cell-based and do not provide a long-term female germ cell system with well-defined germline properties. Germ cell identity is generally assessed by integrating cellular characteristics with conserved molecular markers. Genes such as *Vasa*, *Nanos*, *Piwi*, *Dazl*, and *Dnd1* are widely associated with germ cell development and maintenance across vertebrates, and are commonly used for germline identification [21,28]. Among them, *Vasa* is a conserved DEAD-box RNA helicase expressed throughout multiple stages of germ cell development [29,30], whereas *Nanos* family members participate in the survival, maintenance, and differentiation of early germ cells [31]. In addition to marker-based characterization, RNA sequencing (RNA-seq) enables transcriptome-wide comparison between germ and somatic cells, and facilitates identification of candidate pathways involved in germ cell maintenance and differentiation [32,33]. Combining these approaches therefore provides a more comprehensive framework for evaluating newly established germ cell cultures and exploring their underlying regulatory mechanisms.

In this study, immature Japanese eel ovaries were used to establish a long-term oogonial stem cell-like cell line, designated *A. japonica* oogonial stem cell-like cell line (AjOSCL). Its cellular, genetic, and germline characteristics were evaluated through morphological and growth analyses, chromosome examination, and detection of germline-associated markers. RNA-seq was further performed to characterize its transcriptional profile and identify candidate pathways and genes potentially associated with germ cell maintenance. The establishment of AjOSCL provides a stable in vitro model for investigating the maintenance and differentiation of Japanese eel oogonia and lays a foundation for further studies of female germ cell biology and reproductive biotechnology.

## 2. Materials and Methods

### 2.1. Experimental Fish and Identification of Ovarian Developmental Stage

Experimental Japanese eels (*A. japonica*) were provided by Zehui Fisheries Co., Ltd., (Guangze, Nanping, Fujian, China). Fish were transported to the aquaculture facility of the Fisheries College, Jimei University, Xiamen, immediately after purchase, and were held in recirculating freshwater tanks until tissue collection. A total of 15 cultured female individuals were selected, with a mean body length of 28.3 ± 6.8 cm and a mean body weight of 420 ± 60 g. Fish were euthanized by immersion in ice-water slurry for cold anesthesia (without chemical anesthetics) and immediately dissected for ovarian tissue collection. The gonadosomatic index (GSI) was used to preliminarily evaluate gonadal developmental level. GSI was calculated using the formula: GSI (%) = Wg/Wb × 100, where Wg is the gonad weight and Wb is the body weight. In addition, part of the ovarian tissue was used for histological analysis to determine the developmental stage of the ovary and composition of oogonia and oocytes at different stages, and to identify immature ovaries suitable for subsequent cell isolation and culture. Ovarian tissues were fixed in 4% paraformaldehyde (PFA) (Sangon Biotech, Shanghai, China) at 4 °C for 24 h, dehydrated through a graded ethanol series (Sangon Biotech, Shanghai, China), embedded in paraffin wax, sectioned at 5 μm, and stained with hematoxylin and eosin (H&E) (Sangon Biotech, Shanghai, China). Oocyte developmental stages were determined based on previously described histological characteristics of oogenesis in Japanese eel [34]. Stage II oocytes were defined as previtellogenic (perinucleolar) oocytes characterized by a relatively large germinal vesicle, multiple nucleoli located predominantly along the nuclear periphery, basophilic cytoplasm, and the absence of obvious yolk globules. All animal experiments were approved by the Experimental Animal Ethics Committee of Jimei University (Animal Ethics No. 2021-4, approved on 11 September 2024) and were conducted in accordance with relevant animal welfare and ethical norms.

### 2.2. Ovarian Cell Isolation and Germ Cell Enrichment

Under sterile conditions, ovarian tissue was dissected and thoroughly rinsed with sterile PBS to remove residual blood and tissue debris. The ovary was cut into small fragments and enzymatically digested using type IV collagenase (Sigma-Aldrich, Darmstadt, Germany) combined with DNase I (Sigma-Aldrich, Darmstadt, Germany), with trypsin (Sangon Biotech, Shanghai, China) additionally used if necessary. Digestion was carried out at 25–28 °C for 0.5–1 h with intermittent gentle mixing to promote full tissue dissociation while minimizing mechanical damage. After digestion, the enzyme reaction was terminated, and the resulting cell suspension was sequentially filtered through 100-μm and 40-μm cell strainers (Sangon Biotech, Shanghai, China) to remove incompletely digested tissue clumps and large cell aggregates. The filtrate was centrifuged and resuspended, and cell viability was assessed using the trypan blue exclusion method; cells with viability greater than 90% were used for subsequent culture. To reduce interference from ovarian somatic cells in germ cell culture, the differential adhesion method was first used to remove rapidly adherent fibroblast-like cells and other somatic cells, followed by Percoll (Pharmacia, Stockholm, Sweden) density-gradient centrifugation to further enrich germ cells. The Percoll gradient was set at 20%, 40%, and 60%. Because most oocytes had been eliminated by the 40-μm filtration and germline stem cells are both larger and denser than most ovarian somatic cells, they sedimented into the lower-density region and were collected at the 20–40% interface, whereas the 60% layer, containing high-density debris and residual somatic cells, was discarded. Further selection was performed based on cell size and morphological characteristics when necessary [21]. The enrichment efficiency was comprehensively evaluated by cell viability, cell morphology, and expression of germ cell-related marker genes.

### 2.3. Establishment, Passaging, and Cryopreservation of the Cell Line

The enriched cells were seeded at a density of 1 × 10^5^ cells/mL in complete medium based on Leibovitz’s L-15 (Gibco, Grand Island, NK, USA), supplemented with 10–15% fetal bovine serum (FBS) (ZETA life, San Francisco, CA, USA), 20 ng/mL stem cell factor (SCF) (PeproTech, Cranbury, NJ, USA), 10 ng/mL basic fibroblast growth factor (β-FGF) (PeproTech, Cranbury, NJ, USA), 10 ng/mL leukemia inhibitory factor (LIF) (PeproTech, Cranbury, NJ, USA), and 1% penicillin–streptomycin mixture (Sangon Biotech, Shanghai, China), and cultured at 25 °C [27]. Following 2–5 days of primary culture, the medium was changed according to cell adherence and growth status, and the first passage was performed (0.25% trypsin digestion) once the cells reached approximately 70–80% confluence. Cells were subsequently passaged at a split ratio of 1:2–1:3, re-seeded at 2 × 10^5^ cells/mL in a 25 cm^2^ culture flask, and subcultured at interval of 3–5 days. Early (P1–P10), middle (P11–P25), and late (>P25) passages were selected to compare cell stability. Cells in logarithmic growth phase at 80–90% confluence were cryopreserved, and then the cell viability, adherence, and growth-recovery capacity were assessed to evaluate the freeze–thaw performance of the cell line after thawing. A stable cell line was defined as cells maintaining molecular characteristics for ≥6 months and ≥30 passages.

### 2.4. Determination of Cell Morphology and Proliferative Capacity

Cell morphology, adherence, and proliferation status were observed using an inverted microscope (Leica, Wetzlar, Hesse, Germany), and morphological changes at different culture times and passage stages were recorded. For morphological characterization, cells at logarithmic growth phase were subjected to HE (Hematoxylin–Eosin) staining to examine the nuclear-to-cytoplasmic ratio and cytoplasmic staining characteristics. To determine growth kinetics, cells were seeded at 5 × 10^4^ cells/mL in 24-well plates, and every 24 h over a consecutive 7-day period, six wells were trypsinized and counted per time point to construct growth curves. The population doubling time (PDT) was subsequently calculated based on cell numbers during the logarithmic growth phase. Colony-formation assays were performed using a low-density seeding method, and sustained clonal proliferative capacity was further evaluated through single-cell limited dilution, clonal expansion, and secondary colony formation [28]. In addition, PI staining combined with flow cytometry was used to analyze the proportions of cells in G0/G1, S, and G2/M phases.

### 2.5. Karyotype and Genetic Stability

Logarithmic-phase cells were treated with colchicine at a final concentration of 0.2 μg/mL for 3–4 h to arrest cells at metaphase [35]. After collection, cells were subjected to hypotonic treatment with 0.075 mol/L KCl and fixed with a 3:1 (*v*/*v*) methanol–glacial acetic acid fixative. Following dropping onto slides and Giemsa staining, well-spread metaphase chromosome spreads were selected for observation and counting under the microscope, and the distribution of chromosome numbers and the proportion of diploid cells were determined. Chromosome composition at different passage stages was compared to evaluate genetic stability during long-term passaging. In addition, genomic DNA was extracted, and the mitochondrial cytochrome c oxidase subunit I (*COI*) gene was amplified and sequenced; the obtained sequence was aligned with the Japanese eel reference sequence to confirm the species origin of the cell line and exclude cross-contamination by heterologous cells.

### 2.6. Detection of Germline-Related and Pluripotency-Related Markers

Semi-quantitative PCR and immunofluorescence were used to examine the germline-related and pluripotency-related molecular characteristics of the cell line. For semi-quantitative PCR, the total RNA was extracted from AjOSCL and AjOTCL cells using a Total RNA Isolation Kit (Sangon Biotech, Shanghai, China) following the manufacturer’s instructions. RNA quality was assessed with a NanoDrop 2000 spectrophotometer (Thermo Fisher Scientific, Wilmington, DE, USA) and agarose gel electrophoresis. For each sample, 1.0 μg of total RNA was reverse-transcribed into first-strand cDNA using a first-strand cDNA synthesis kit (Thermo Fisher Scientific, Waltham, MA, USA), and the resulting cDNA was stored at −20 °C for subsequent gene cloning and expression analysis. Semi-quantitative PCR was performed under the following conditions: initial denaturation at 94 °C for 5 min; 25 cycles of denaturation at 94 °C for 10 s, annealing at 60 °C for 15 s, and extension at 72 °C for 10 s; and a final extension at 72 °C for 5 min. Germline-related marker genes included *Vasa*, *Nanos2* and *Nanos3* [30,31]; undifferentiation-related markers included *Oct4*, *Nanog*, and *Sox2* [36,37], and ovarian somatic cell-related marker genes *Foxl2* and *Sox3* [38,39] were also examined to assist in determining cell lineage characteristics. And gene specific primers were designed using Primer Premier 5.0 (Premier Biosoft Interpairs, Palo Alto, CA, USA) (Table S1).

For immunofluorescence, cells grown on coverslips were fixed in 4% PFA in PBS for 15 min at room temperature (RT), washed three times with PBS, and permeabilized with 0.5% Triton X-100 (Sangon Biotech, Shanghai, China) in PBS for 10 min at RT. After blocking with 5% BSA in PBS for 1 h at RT, cells were incubated overnight at 4 °C with primary antibodies diluted in 1% BSA/PBS: anti-Oct4 (ab19857, Abcam; 1:1000), anti-Nanog (ab21624, Abcam, Waltham, MA, USA; 1:1000), anti-Nanos2 (ab70000, Abcam, Waltham, MA, USA; 1:500), anti-DDX4/Vasa (ab27591, Abcam, Waltham, MA, USA; 1:100), and anti-Sox2 (ab79351, Abcam, Waltham, MA, USA; 1:200). Cells were then washed three times with PBS (5 min each) and incubated for 1 h at RT in the dark with Alexa Fluor^®^ 488- or 555-conjugated secondary antibodies (Abcam, Waltham, MA, USA) (goat anti-rabbit or goat anti-mouse IgG; 1:1000). After three final PBS washes, nuclei were counterstained with DAPI (Phygene, Fuzhou, Fujian, China; 1:1000), and images were captured in the green (Alexa Fluor^®^ 488) and red (Alexa Fluor^®^ 555) channels under a fluorescence microscope (Leica, Wetzlar, Hesse, Germany) to examine the expression and subcellular localization of the target proteins, followed by fluorescence signal analysis. The specificity of the antibodies used for immunofluorescence staining was further validated by Western blotting (Figure S1). In addition, alkaline phosphatase staining was performed using NBT/BCIP Stock Solution (Roche, Basel, Switzerland; 1:100) to evaluate the pluripotency-related phenotype. All above assays were performed in three independent replicates.

### 2.7. Transcriptome Sequencing Analysis

AjOSCL and the Japanese eel ovarian somatic cell line AjOTCL [27] were collected, with three biological replicates per group. Total RNA was extracted described in Section 2.6, and RNA quality was assessed using an Agilent 2100 Bioanalyzer (Agilent Technologies, Santa Clara, CA, USA); only RNA samples with an RNA Integrity Number (RIN) ≥ 7.0 were used for library preparation. Sequencing libraries were constructed from 1 μg of total RNA using the TruSeq^TM^ RNA Library Prep Kit v2 (Illumina, San Diego, CA, USA) following poly(A) enrichment, and non-stranded-specific libraries were sequenced on an Illumina NovaSeq 6000 platform (Illumina, San Diego, CA, USA) with paired-end 150-bp (PE150) reads. Raw reads were assessed using FastQC (v0.11.9) and trimmed using Trimmomatic (v0.39) to remove adapter sequences and low-quality bases. Clean reads were aligned to the Japanese eel reference genome (GCA_025169545.1) [40] using HISAT2 (v2.2.1). Gene-level read counts were obtained using FeatureCounts (v2.0.3), and differential expression analysis was performed using DESeq2 (v1.36.0); genes with |log2FC| ≥ 1 and an adjusted *p*-value (Padj) < 0.05 were considered differentially expressed. Gene Ontology (GO) (https://geneontology.org/, accessed on 1 January 2026) and Kyoto Encyclopedia of Genes and Genomes (KEGG) (https://www.genome.jp/kegg/, accessed on 1 January 2026) enrichment analyses were conducted using clusterProfiler (v4.4.4) and KOBAS (v3.0.3), respectively. Particular attention was paid to genes and pathways associated with germ cell development, undifferentiated-state maintenance, oocyte development, and associated signal regulation, including Cell cycle, TGF-β, and Wnt signaling [41], to characterize the transcriptional features of AjOSCL and explore molecular pathways potentially associated with its germline-related properties.

### 2.8. Data Processing

Unless otherwise specified, all cytological experiments were performed in at least three independent replicates, with three wells within the same independent experiment serving as technical replicates, and results are expressed as mean ± standard deviation (mean ± SD). Statistical analyses were performed using SPSS 27.0 software. Normality and homogeneity of variance were tested before analysis. Comparisons between two groups were conducted using the independent-samples *t*-test when parametric conditions were met, or the Mann–Whitney U test otherwise; comparisons among multiple groups were conducted using one-way or two-way analysis of variance (ANOVA) depending on the experimental design. Data at continuous time points, such as growth curves, were analyzed using repeated-measures ANOVA or mixed-effects models. For multiple comparisons, the Bonferroni method or FDR correction was applied, and *p* < 0.05 was considered statistically significant. Figures were generated using GraphPad Prism 8 (v8.4.3) and laid out using Adobe Illustrator 2023 (v27.9).

## 3. Results

### 3.1. Characterization of Immature Ovarian Tissue as a Source of Oogonia in Japanese Eel

**Figure 1:**
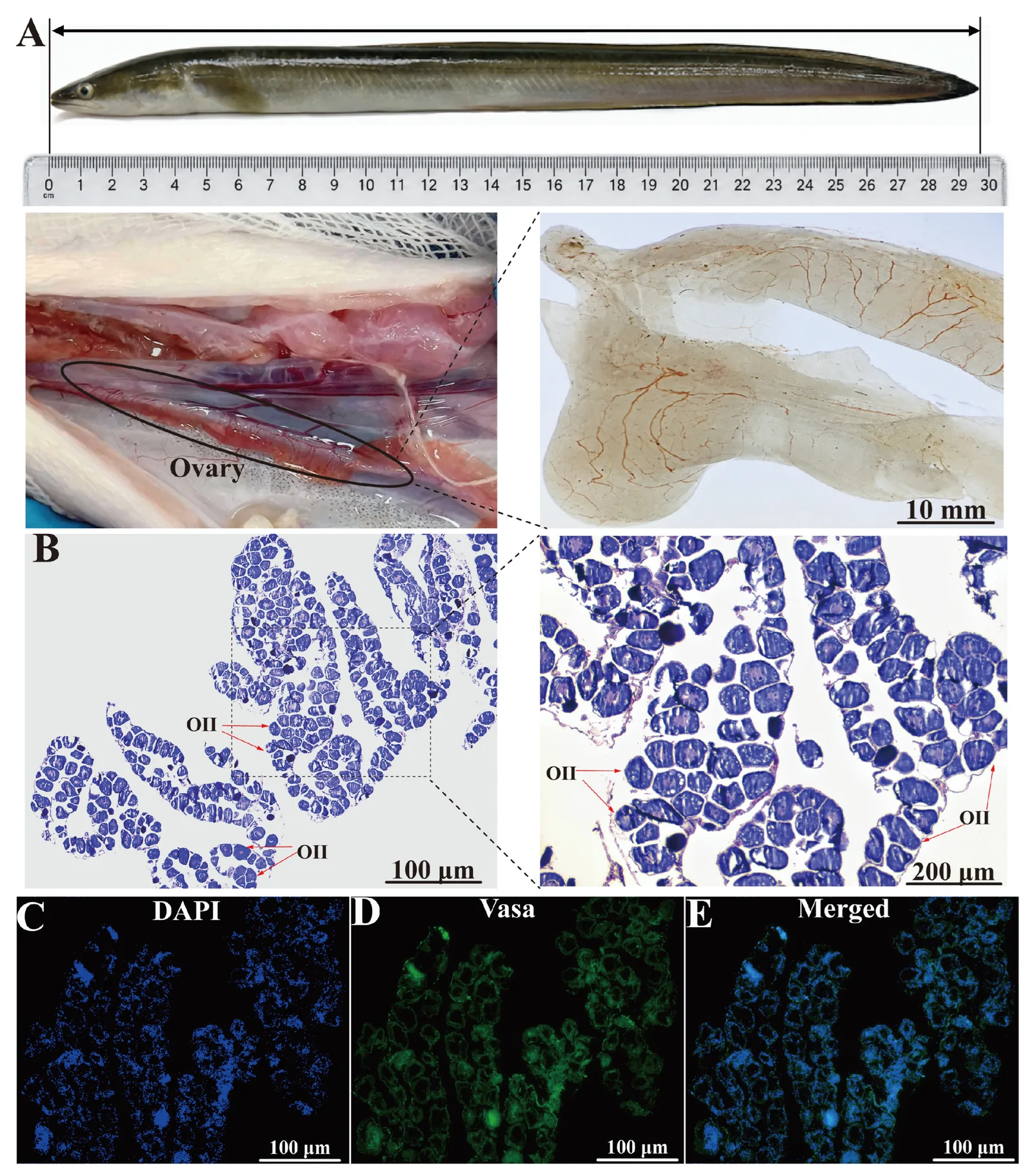
Morphological and histological characteristics of immature ovaries of *A. japonica*. (**A**) Gross morphology of the Japanese eel and its ovary. The ovary appeared multiple-lobed (sheet-like) and translucent (black circle and magnified view). (**B**) HE staining of an ovarian section, showing abundant early stage II oocytes (OII). Stage II oocytes were defined as previtellogenic (perinucleolar) oocytes characterized by a relatively large germinal vesicle, multiple nucleoli distributed predominantly along the nuclear periphery, basophilic cytoplasm, and the absence of obvious yolk globules. The red arrow indicates stage II oocytes (OII) of the ovary. (**C**–**E**) Immunofluorescence localization of the germline marker Vasa in immature ovaries. (**C**) DAPI-stained nuclei (blue). (**D**) Vasa-positive signals (green) are broadly distributed and localized in early oocytes, indicating that the ovarian tissue is rich in germ cells. (**E**) Merged image of DAPI and Vasa staining.

The gonadosomatic index (GSI) of the experimental Japanese eels was 0.18 ± 0.04%, a value well below that reported for maturing silver-phase females (0.3–0.9%) [42], confirming that the sampled ovaries remained at an immature, previtellogenic stage. Grossly, the ovaries were multiple-lobed (sheet-like) and translucent, and their histological characteristics were consistent with the morphology of immature ovaries (Figure 1A). Histological examination showed that the ovaries were at an early developmental stage and were predominantly composed of stage II previtellogenic (perinucleolar) oocytes, characterized by a prominent germinal vesicle, peripheral nucleoli, basophilic cytoplasm, and the absence of obvious yolk accumulation (Figure 1B). Immunofluorescence analysis revealed that Vasa was broadly expressed throughout the immature ovary, and its ubiquitous distribution confirmed the germline identity of these cells rather than a somatic cell origin (Figure 1C–E). Collectively, these morphological and molecular data indicate that the immature ovaries consisted predominantly of early stage II oocytes, and likely harbored a higher proportion of germline stem cells (GSCs) than more mature ovaries, rendering them well suited as a tissue source for subsequent germ cell isolation and culture.

### 3.2. Isolation and Enrichment of Germline Cells for Long-Term Culture

**Figure 2:**
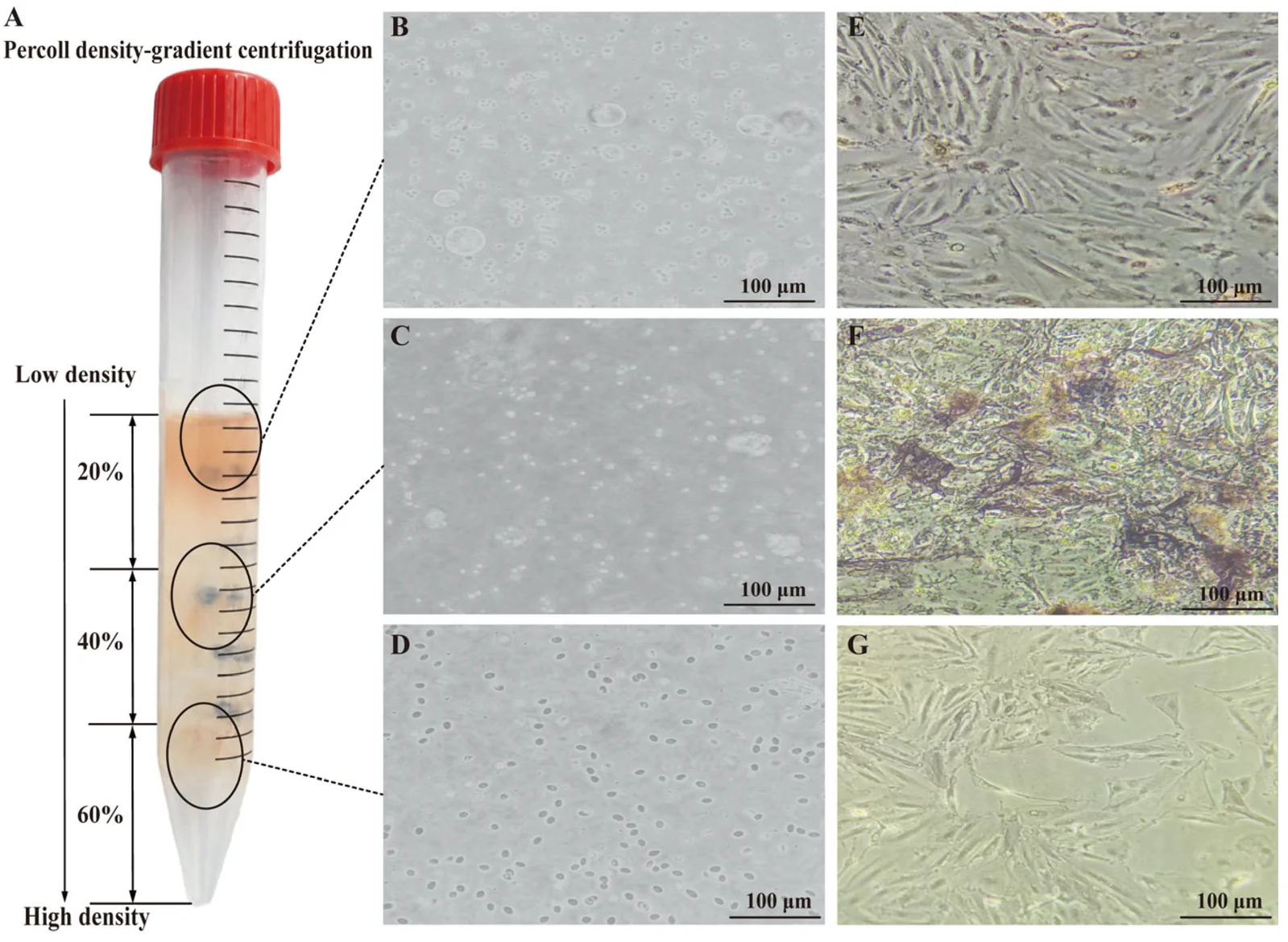
Isolation and enrichment of ovarian germ cells from *A. japonica* via Percoll density-gradient centrifugation. (**A**) Schematic diagram showing the enrichment of ovarian cells using a Percoll density gradient (20%, 40%, and 60%). (**B**–**D**) Morphology of cells collected from different Percoll interface layers: (**B**) the 20% layer contains large-diameter oocytes and cellular debris; (**C**) the 40% layer is enriched with undifferentiated germ cells; (**D**) the 60% layer predominantly consists of erythrocytes and small cellular fragments. (**E**–**G**) Alkaline phosphatase (AP) staining of cells from the 20%, 40%, and 60% Percoll layers after 5 days of culture, respectively. (**E**) Partial AP-positive (blue/purple precipitate) cells are observed in the 20% layer; (**F**) the 40% layer exhibits the highest abundance of AP-positive cells; (**G**) almost no AP-positive cells are detected in the 60% layer.

The cell population obtained after primary digestion exhibited obvious heterogeneity, comprising round cells, fibroblast-like spindle-shaped cells, and a small number of irregular cells. After enrichment by differential adhesion and Percoll density-gradient centrifugation, the proportion of round cells was significantly increased (Figure 2A). After collecting and observing cells from each fraction, a small number of oocytes were present in the 20% layer (Figure 2B), and the most cells aggregated in the 40% separation layer (Figure 2C), while the 60% fraction contained numerous somatic cells and blood cells (Figure 2D). Cells before separation and those from the 40% fraction after separation were separately transferred to culture plates treated with 0.1% gelatin; after cells adhered and were cultured for 2 days, alkaline phosphatase (AP) staining was performed (Figure 2E–G). Both cells directly dissociated from the ovary into single cells and cells from the 20–40% Percoll fraction exhibited strong alkaline phosphatase activity, indicating their possible stem cell characteristics and meeting the requirements for subsequent culture.

### 3.3. Establishment of a Long-Term Cultured Oogonial Stem Cell-like Line from Japanese Eel

**Figure 3:**
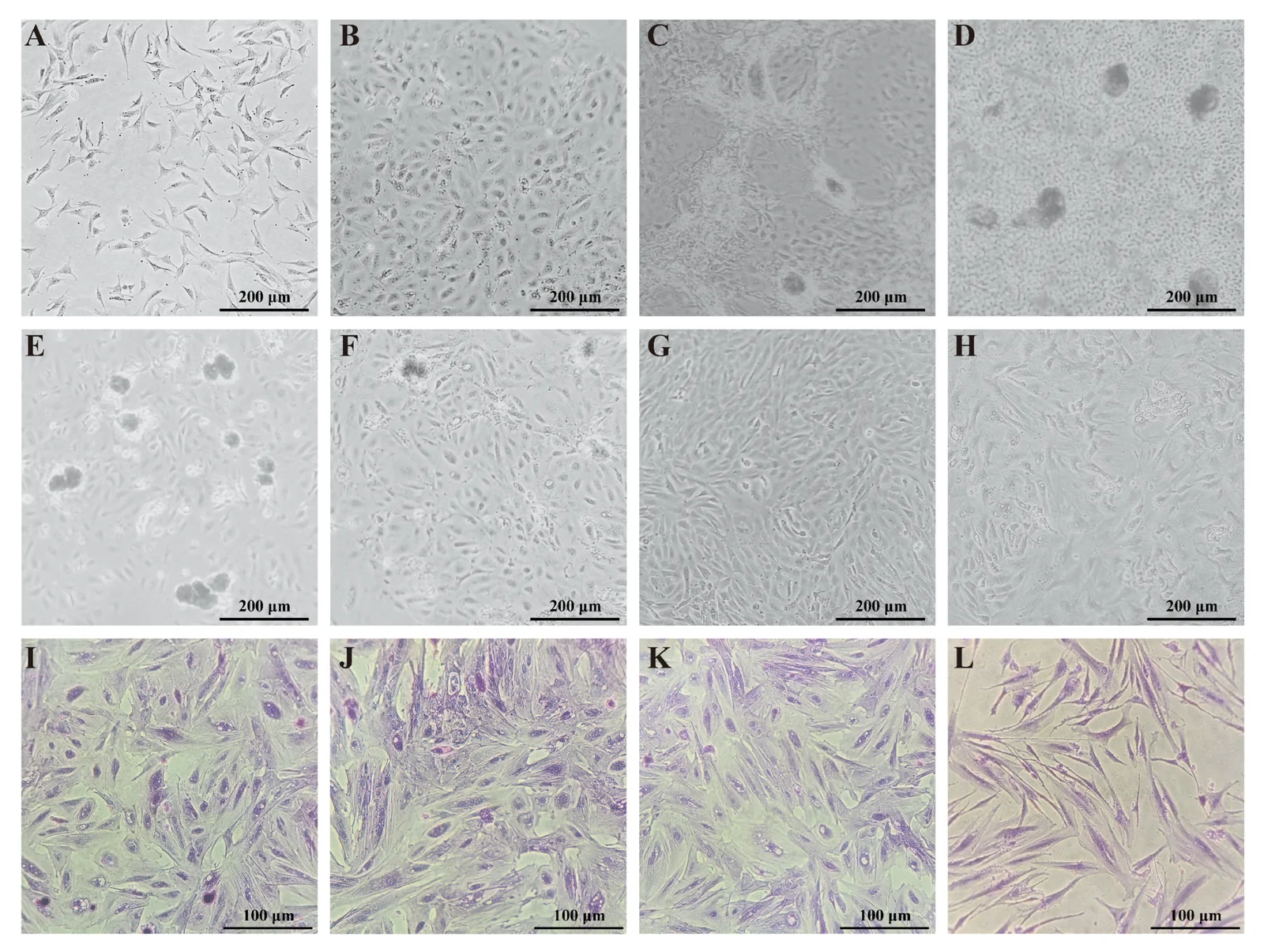
Establishment and morphological characterization of *A. japonica* oogonial stem cell-like line AjOSCL. (**A**–**D**) Morphology of primary cells at days 2, 7, 14, and 21 of initial culture, respectively. (**E**–**G**) Morphology of passage 10 (P10), P20, and P30 cells, respectively. (**H**) Cell morphology after cryopreservation and thawing for 48 h. (**I**–**K**) HE-stained morphological features of cells at different passage stages (P5, P15, and P25). (**L**) HE-stained morphology of the Japanese eel ovarian somatic cell line AjOTCL.

The cells in 40% layer were cultured in the optimization L-15 medium. Some cells began to adhere within 48 h of primary culture (Figure 3A), and the density of most cells increased to 90% after 7 days (Figure 3B). Distinct clone-like cell colonies formed by day 14 (Figure 3C), and the colonies were mainly composed of tightly arranged round or short spindle-shaped cells with a diameter of approximately 100–300 μm (Figure 3D). The first passage was performed on day 15 at a split ratio of 1:2, after which the cells gradually established a stable growth state with a passage interval maintained at 5–7 days (Figure 3E–G). The cell morphology after cryopreservation and subsequent recovery showed no significant difference from that before (Figure 3H). The cell line had been continuously passaged for more than 35 generations and was designated oogonial stem cell-like cell line of *A. japonica* (AjOSCL).

Cell morphology remained generally stable across different passage stages. P1–P10 cells were mainly small round or ovoid with a high nuclear-to-cytoplasmic ratio, and some cells exhibited distinct nucleoli (Figure 3I); P15–P25 cells were still predominantly round and short spindle-shaped, with a small number of polygonal cells (Figure 3J); in P30 and above, the proportion of long spindle-shaped cells increased slightly, but round and short spindle-shaped cells still predominated (Figure 3K). Overall, the basic morphological characteristics of P5 and P30 cells remained consistent, and no obvious cellular senescence were observed during long-term passaging. Compared with the ovarian somatic cell AjOTCL, AjOSCL exhibited more obvious characteristics of small size, high nuclear-to-cytoplasmic ratio, and clone-like growth (Figure 3L).

### 3.4. Growth Characteristics and Self-Renewal Capacity of AjOSCL

**Figure 4:**
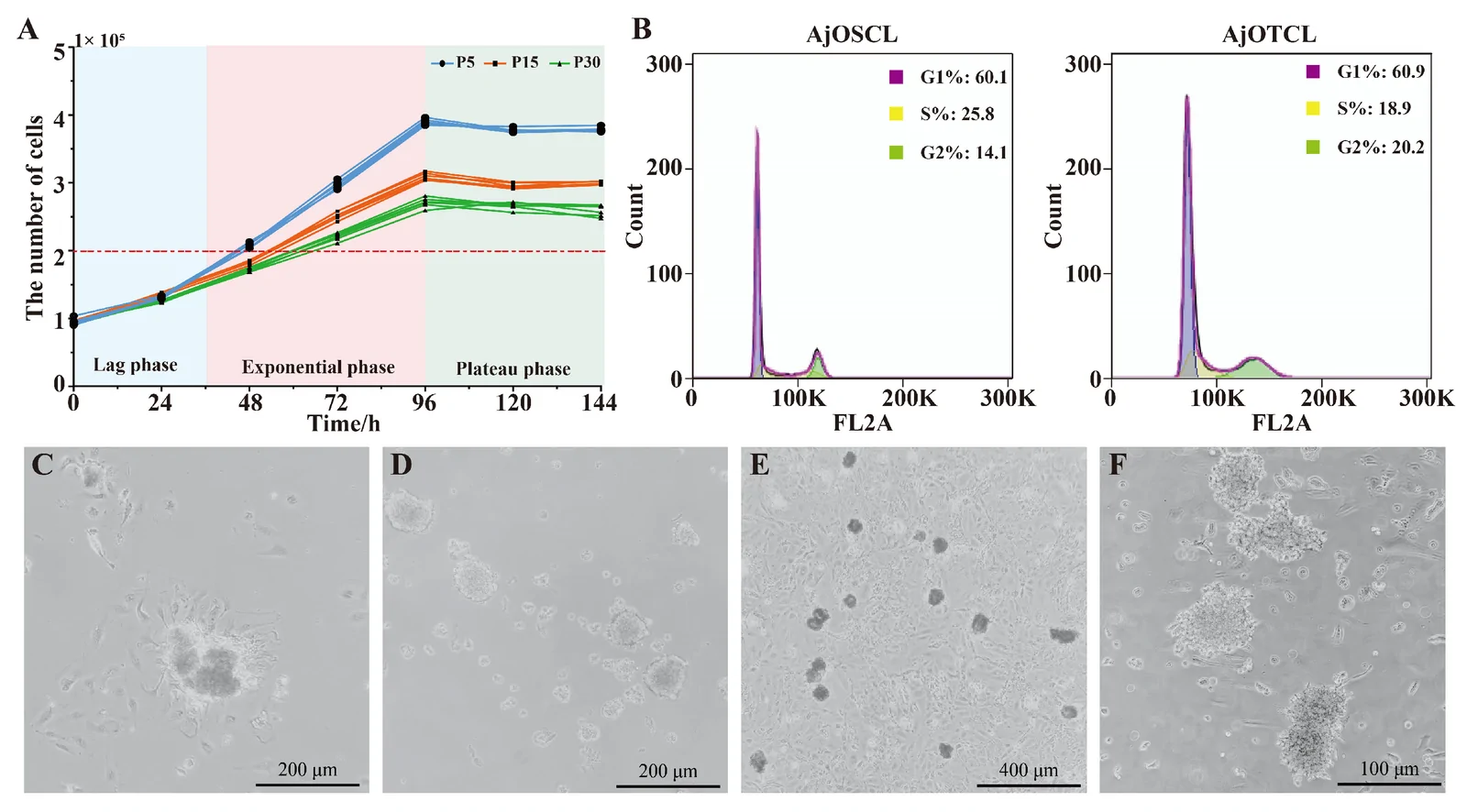
Growth characteristics and cell cycle analysis of AjOSCL. (**A**) Cell growth curves. Growth kinetics of cells at different passages (P5, P15, and P30) were monitored over 144 h. The growth profile is divided into the lag phase (0–36 h), exponential phase (36–96 h), and plateau phase (96–144 h). The red dashed line indicates the population doubling time (PDT) during the exponential growth phase. (**B**) Cell cycle analysis. Flow cytometry analysis of cell cycle distribution in AjOSCL and AjOTCL groups. (**C**–**F**) Morphological assessment of clone formation. Representative micrographs showing (**C**) single cells inoculated after dilution, (**D**) clone proliferation at 48 h post-inoculation, (**E**) clone growth at 5 days after secondary inoculation, and (**F**) a magnified view of the clone at 5 days after secondary inoculation.

Growth-curve analysis showed that P5, P15, and P30 AjOSCL cells all exhibited typical S-shaped growth curves, sequentially undergoing lag, logarithmic growth, and plateau phases. The population doubling times of P5, P15, and P30 cells were 42.5 ± 1.1 h, 50.2 ± 1.3 h, and 55.6 ± 2.1 h, respectively. Although the population doubling time showed a numerical trend toward prolongation with increasing passage number, the differences among passages were not statistically significant (*p* > 0.05), indicating that AjOSCL maintained relatively stable proliferative capacity during long-term passaging (Figure 4A). Cell cycle analysis demonstrated that AjOSCL significantly increased the proportion of S-phase cells while reducing the G2/M-phase population compared to the AjOTCL group (*p* < 0.05), suggesting that it promotes DNA synthesis and accelerates cell cycle progression (Figure 4B). Low-density colony-formation assays showed that AjOSCL could form dense cell colonies with relatively clear boundaries (Figure 4C–F). Following limited dilution cloning, 18 colonies derived from single cells were obtained, of which 8 were successfully expanded. Among these, 6 clones exhibited a morphology similar to that of the primary germ cell culture and sustained proliferation. One representative clone with stable morphology and robust long-term growth was selected for continuous passaging, leading to the establishment of the AjOSCL cell line. Clonality was ensured by limiting dilution seeding in 96-well plates (1 clone/well), and plates were examined microscopically 24 h after seeding to confirm wells containing exactly one cell. These results indicated that AjOSCL possesses relatively stable clonal proliferation and continuous colony-forming capacity, providing cytological evidence for evaluating its long-term self-renewal potential.

### 3.5. Molecular Validation and Characterization of AjOSCL

**Figure 5:**
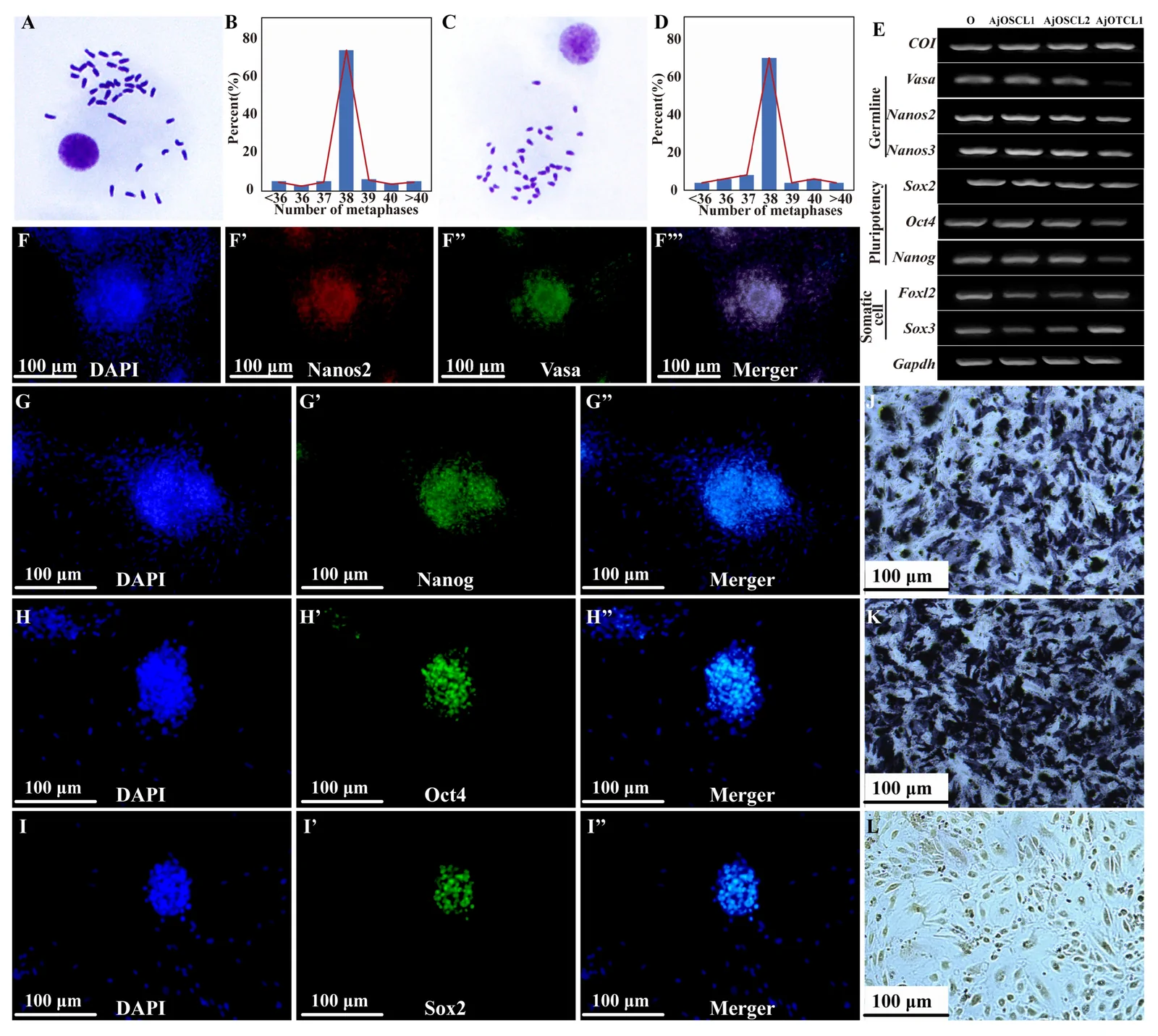
Characterization of genetic stability, gene expression, and germline-specific markers in the AjOSCL. (**A**–**D**) Karyotype analysis of AjOSCL. Representative metaphase chromosomes (**A**,**C**) and frequency distribution of chromosome numbers (**B**,**D**) showing a modal number of 2*n* = 38. The red line in the figure represents the median number of chromosomes during each phase of cell division. (**E**) RT-PCR analysis of germline-related (*Vasa*, *Nanos2*, *Nanos3*), pluripotency-related (*Sox2*, *Nanog*, *Oct4*) genes, and ovarian somatic cell-related genes *Foxl2* and *Sox3*. *COI* and *Gapdh* were used as species and internal control, respectively. O represents Japanese eel ovarian tissue. AjOSCL1 and AjOSCL2 represents AjOSCL at P15 and P30, respectively. (**F**–**F‴**) Immunofluorescence co-localization of Nanos2 (green) and Vasa (red) in cell colonies. (**G**–**I″**) Immunofluorescence staining of pluripotency markers Nanog (**G’**), Oct4 (**H’**), and Sox2 (**I’**). Nuclei were counterstained with DAPI (blue). (**J**,**K**) AP staining of AjOSCL colonies and passaged cells at P5 (**J**) and P15 (**K**), indicating strong AP activity. (**L**) AP staining of AjOTCL, indicating none AP activity.

Mitochondrial *COI* sequencing of AjOSCL showed more than 99% identity with the Japanese eel sequence in the NCBI database, and all BLAST results matched *A. japonica*, confirming that the cell line originated from *A. japonica* with no obvious heterologous cell contamination. Chromosome karyotype analysis showed that the chromosome number of AjOSCL was predominantly 2*n* = 38, with metaphase spreads containing 38 chromosomes accounting for approximately 72% of the counted cells, indicating a diploid-dominant state (Figure 5A–D). No obvious large-scale chromosomal structural abnormalities were observed in long-term passaged cells, indicating that AjOSCL possesses good chromosomal stability during continuous passaging.

The semi-quantitative PCR results revealed markedly stronger expression of the germ cell-related gene *Vasa* and the pluripotency-related genes *Nanog* and *Oct4* in AjOSCL, whereas their expression was weak or undetectable in the ovarian somatic cell line AjOTCL. *Nanos2*, *Nanos3*, *Sox2*, and the ovarian somatic cell-related genes *Foxl2* and *Sox3* were detectable in both cell lines, with no obvious differences in expression patterns observed between AjOSCL and AjOTCL (Figure 5E). Immunofluorescence further showed that Vasa and Nanos2 proteins exhibited a granular distribution in the colonies of AjOSCL (Figure 5F–F‴), and Nanog (Figure 5G–G″), Oct4 (Figure 5H–H″) and Sox2 (Figure 5I–I″) proteins also showed strong positive signals in the colonies. Results obtained by different detection methods were largely consistent, indicating that AjOSCL possesses obvious germline-related and pluripotency-related molecular characteristics. In addition, both P5 and P15 AjOSCL exhibited strong positive signals of alkaline phosphatase (AP) staining, which mainly distributed in the cytoplasm and cell-membrane regions (Figure 5J–K), with no obvious changes in AP staining intensity or the proportion of positive cells between passages, whereas AjOTCL showed weak or no obvious positive AP signals (Figure 5L). These results further indicated that AjOSCL can maintain a relatively stable pluripotency-related phenotype during long-term culture.

### 3.6. Transcriptome Sequencing and Functional Enrichment Analysis of DEGs in AjOSCL

**Figure 6:**
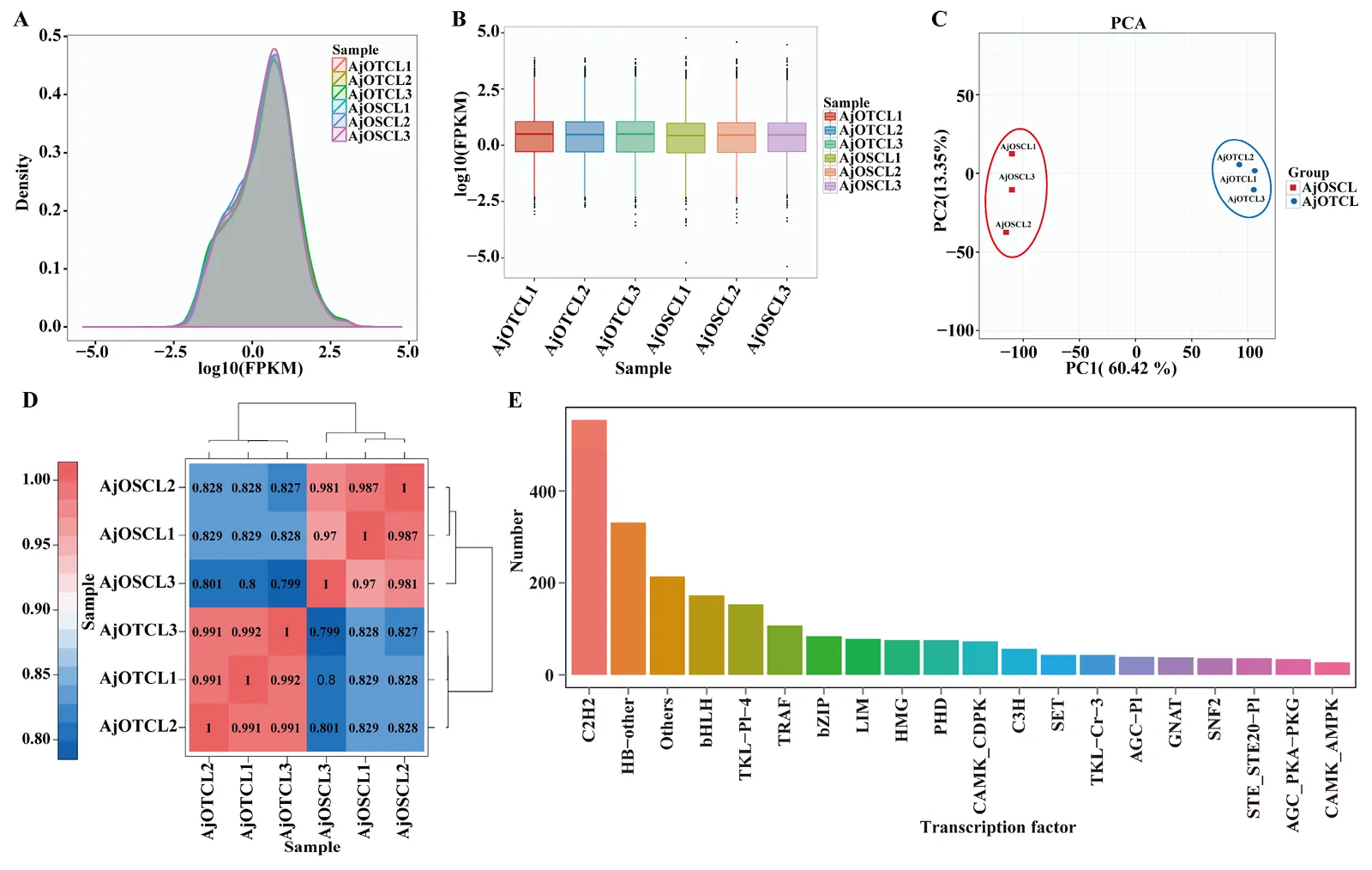
Transcriptome profiling, quality assessment, and transcription factor prediction of AjOSCL and AjOTCL. (**A**) Density plot illustrating the distribution of gene expression levels (log10 FPKM) across all AjOSCL and AjOTCL samples. (**B**) Boxplot showing the statistical distribution of gene expression levels (log10 FPKM) for each biological replicate. (**C**) Principal Component Analysis (PCA) plot of the transcriptome data, demonstrating clear spatial separation and distinct clustering between AjOSCL and AjOTCL groups. (**D**) Heatmap of Pearson correlation coefficients among the samples. The color gradient indicates the strength of correlation, confirming high intra-group reproducibility. (**E**) Bar chart displaying the classification and absolute number of predicted genes assigned to various transcription factor families in the ovarian cells.

To elucidate the transcriptional molecular characteristics of AjOSCL, RNA-seq analysis was performed on AjOSCL and control AjOTCL, utilizing three biological replicates per group. Sequencing generated a total of approximately 36.87 Gb of raw data, with each sample yielding approximately 5.92 Gb of clean data. The Q30 base percentage exceeded 94.70%, and the mapping rate of clean reads to the Japanese eel reference genome was approximately 85%, confirming that all quality metrics met the stringent requirements for downstream transcriptome analysis (Table S2). Gene expression density and boxplots demonstrated highly stable and consistent overall expression distributions across samples, alongside stage-specific differences in the proportion of highly expressed genes (Figure 6A,B). Sample correlation analysis and principal component analysis (PCA) demonstrated tight clustering of the three biological replicates within each group (Figure 6C). Furthermore, AjOSCL and AjOTCL samples formed distinct, well-separated clusters at the transcriptome level, reflecting high intra-group reproducibility and markedly divergent global gene expression patterns between the two cell types (Figure 6D). Transcription factor (TF) prediction based on the transcriptome data revealed a rich repertoire of TF families in both oogonial stem cells and ovarian somatic cells (Figure 6E). Notably, the C2H2, bHLH, bZIP, HB, and AP2/ERF families accounted for the majority of predicted TFs, suggesting that these regulatory networks may play crucial roles in cell fate determination and ovarian tissue development.

**Figure 7:**
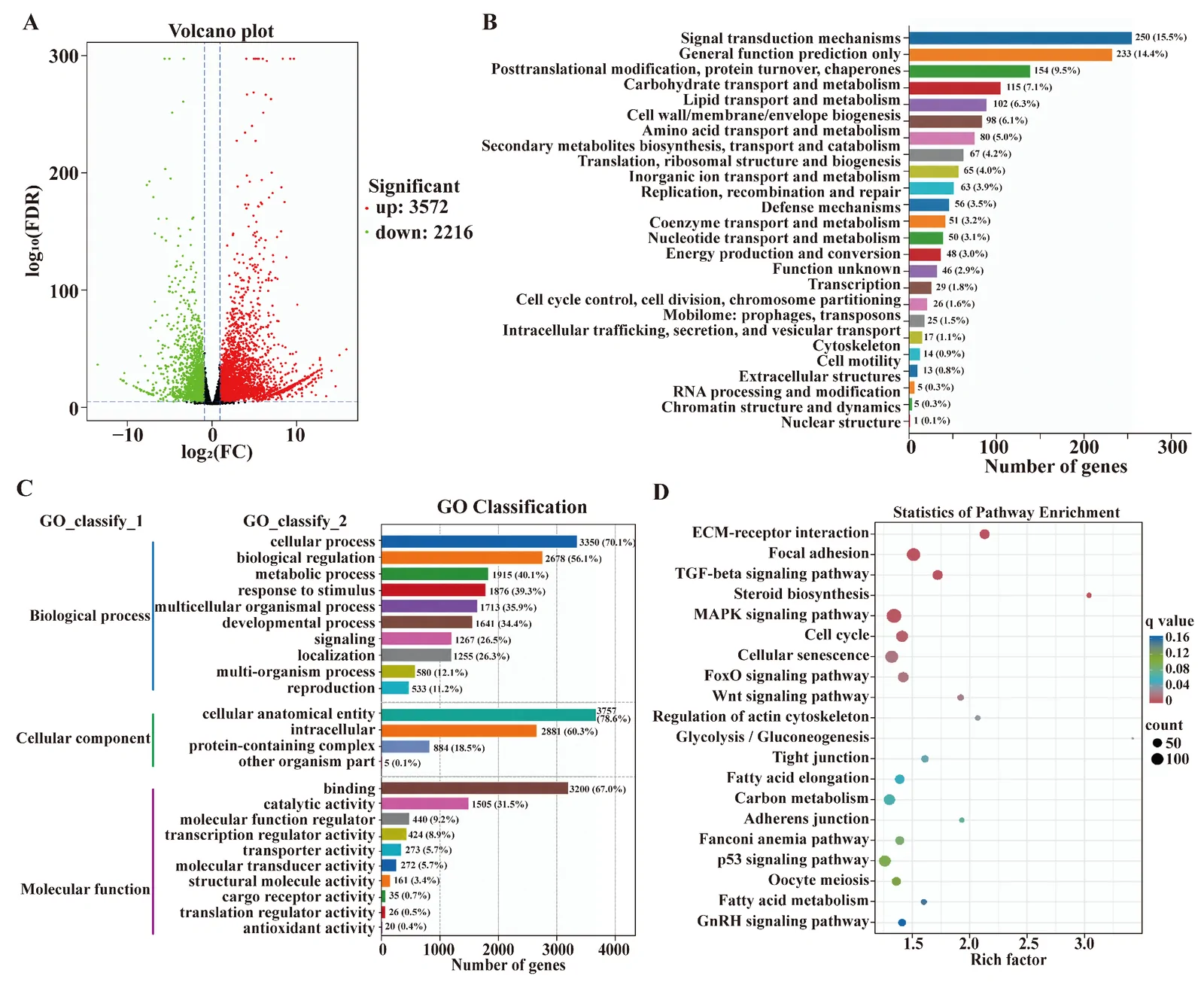
Transcriptomic profiling and functional enrichment analysis of differentially expressed genes (DEGs). (**A**) Volcano plot of DEGs. Each point denotes a gene; the *x*-axis indicates the log_2_ (fold change) in expression between two stages (larger absolute values reflect greater expression differences), and the *y*-axis represents the −log_10_(adjusted *p*-value), with higher values indicating more statistically significant differential expression. (**B**) COG functional classification of genes. (**C**) Gene Ontology (GO) functional classification of DEGs categorized into biological process, cellular component, and molecular function. (**D**) Statistics of KEGG pathway enrichment for DEGs. The *y*-axis indicates the pathway name, and the *x*-axis represents the Rich Factor (the ratio of the proportion of DEGs annotated to a specific pathway to the proportion of all genes annotated to that pathway). A larger Rich Factor indicates a higher level of enrichment significance of DEGs in that pathway. The color of the circle corresponds to the *q*-value (the *p*-value adjusted for multiple testing), where a smaller *q*-value indicates more reliable enrichment significance. The size of the circle reflects the number of genes enriched in the pathway.

Using |log_2_FC| ≥ 1 and FDR < 0.05 as screening criteria, a total of 5788 DEGs were identified, of which 3572 were up-regulated and 2216 were down-regulated in AjOSCL relative to AjOTCL (Figure 7A), indicating widespread transcriptional differences between the two cell types.

COG functional classification showed that the DEGs were mainly involved in functional categories such as general function prediction, post-translational modification and degradation of proteins, transcription regulation, translation and ribosomal structure, and energy production and conversion (Figure 7B). In addition, many DEGs were detected in functional categories including cell cycle, signal transduction, membrane transport, and cytoskeleton, suggesting obvious differences between AjOSCL and AjOTCL in proliferation status, cellular structure, and signal regulation.

GO enrichment analysis showed that the DEGs were mainly enriched in biological processes (such as developmental processes, multicellular organism development, tissue development, and regulation of cell differentiation), cellular components (mainly related to the cytoplasm, cell membrane, cell periphery, and extracellular matrix), and molecular functions (protein binding, catalytic activity, and kinase activity) (Figure 7C).

KEGG enrichment analysis further showed that the DEGs were mainly enriched in pathways such as ECM–receptor interaction, focal adhesion, TGF-β, Cell cycle and Wnt signaling pathways, and steroid biosynthesis (Figure 7D). Among these, ECM–receptor interaction and focal adhesion are mainly involved in cell–extracellular matrix interactions, while TGF-β, Cell cycle and Wnt signaling pathways are closely related to cell proliferation, differentiation, and developmental regulation. These results indicated that the transcriptional differences between AjOSCL and AjOTCL mainly involve biological processes such as cell development, proliferation and differentiation, extracellular matrix, and signal regulation.

### 3.7. Transcriptomic Profiling of the Germline Identity and Oogonial Stem Cell-like Potential in AjOSCL

**Figure 8:**
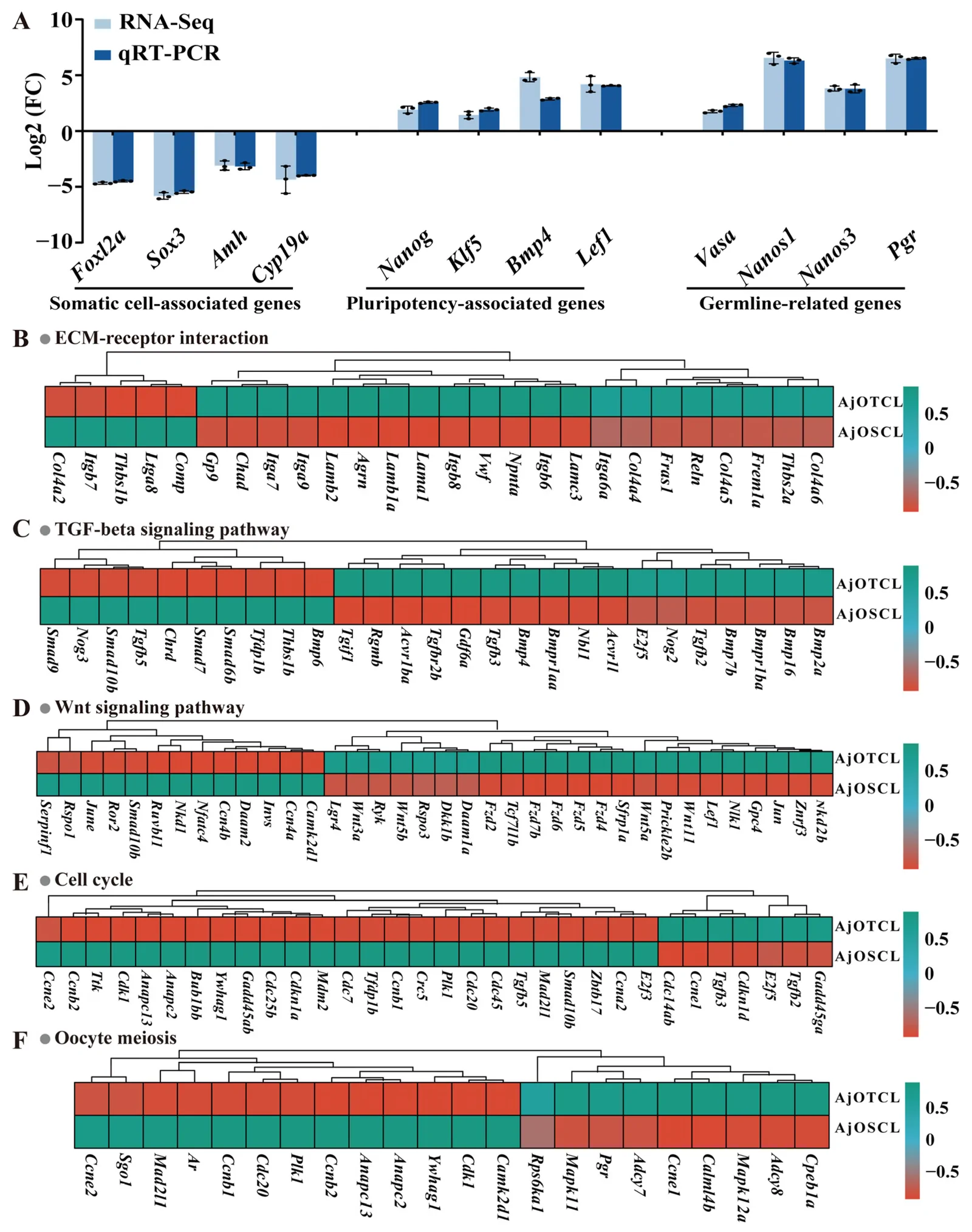
Transcriptomic characterization and validation of differentially expressed genes (DEGs) in AjOSCL. (**A**) Validation of 12 representative DEGs, including somatic cell-associated, pluripotency-related, and germline-related genes, using qRT-PCR and comparison with RNA-Seq data. Data are presented as Mean ± SD. (**B**–**F**) Heatmap analysis of DEGs based on transcriptomic data, showing distinct expression profiles between AjOSCL and AjOTCL in key pathways: (**B**) ECM–receptor interaction; (**C**) TGF-beta signaling pathway; (**D**) Wnt signaling pathway; (**E**) cell cycle; (**F**) oocyte meiosis. The color scale from red to green represents low to high expression levels.

To further characterize the molecular identity of AjOSCL, the expression patterns of germline-associated and ovarian somatic cell-associated genes were compared between AjOSCL and AjOTCL. It revealed that germline-associated genes, including *Vasa*, *Nanos1*, *Nanos3*, and *Pgr*, as well as pluripotency-related genes such as *Nanog*, *Klf5*, *Lef1* and *Bmp4*, were predominantly expressed in AjOSCL. In contrast, ovarian somatic cell-associated markers, including *Foxl2*, *Cyp19a1a*, *Amh* and *Sox3*, exhibited higher expression levels in AjOTCL (Figure 8A). These distinct expression patterns were consistent with the qRT-PCR results.

KEGG pathway enrichment analysis demonstrated that the DEGs in AjOSCL were significantly enriched in pathways associated with germ cell maintenance, stem cell niche regulation, and developmental progression, including ECM–receptor interaction, TGF-β signaling, Wnt signaling, and cell cycle regulation. Within the ECM–receptor interaction and TGF-β pathways, several key niche-associated genes, including *Col4a6*, *Thbs2a*, *Ltgb8*, *Bmp4*, *Tgfb1*, and *Gdf6a*, were highly expressed in AjOSCL (Figure 8B,C), suggesting their potential involvement in maintaining the supportive microenvironment required for germline stem cell characteristics. Furthermore, genes involved in canonical Wnt signaling, a pathway closely associated with stem cell self-renewal and fate maintenance, including *Wnt3a*, *Wnt5a*, *Wnt11*, and *Lef1* (Figure 8D), were significantly upregulated in AjOSCL. In addition, the DEGs were enriched in the cell cycle pathway, including *Cdc14ab*, *Tgfb2*, and *Tgfb3* (Figure 8E), indicating active regulation of proliferation and developmental transitions in AjOSCL.

Importantly, transcriptomic analysis identified significant enrichment of genes associated with the oocyte meiosis pathway, representing a key molecular hallmark of germ cell identity. Several critical regulators involved in meiotic initiation and progression, including *Ccne1*, *Cpeb1a*, and *Pgr*, were specifically upregulated in AjOSCL compared with AjOTCL (Figure 8F). The enrichment of meiotic regulatory programs suggests that AjOSCL retains intrinsic molecular features required for the transition from mitotic proliferation to meiotic differentiation.

## 4. Discussion

### 4.1. Establishment and Characterization of a Long-Term Culture System for AjOSCL

Long-term in vitro maintenance of female germ cells in fish remains considerably more challenging than that of SSCs. The major difficulty lies not merely in sustaining cell viability, but in supporting continuous proliferation while preserving a relatively stable germ cell state outside the native ovarian microenvironment [43,44]. The ovary consists of oogonia, oocytes and their surrounding follicle cells (granulosa cells and theca cells), supporting tissue or stroma, and vascular and nervous tissue [45]. During prolonged culture, these cells can readily overgrow and progressively replace the germ cells [46]. At the same time, OSCs are highly dependent on extracellular matrix components and paracrine signals provided by the ovarian germ cell niche [47,48]. Against this background, the successful isolation and long-term maintenance of AjOSCL from immature Japanese eel ovaries suggest that the combined use of differential adhesion, density-gradient enrichment, and defined growth-factor supplementation effectively addressed two major obstacles in female germ cell culture: enrichment of the target cell population and maintenance of its proliferative state over extended passages.

The supplementation of bFGF, LIF, and SCF may have contributed to the long-term maintenance of AjOSCL [9,35]. FGF signaling is broadly involved in the proliferation and survival of stem and progenitor cells [49], while the SCF/c-Kit axis plays an important role in the survival and expansion of early germ cells in vertebrates [50]. LIF has also been associated with the maintenance of undifferentiated or progenitor-like cell states [37]. AjOSCL maintained relatively stable proliferative and colony-forming capacities during long-term passaging, suggesting that these exogenous signals may partially compensate for the loss of trophic and regulatory cues from the native ovarian niche. This stable in vitro system is particularly valuable for Japanese eel, whose catadromous life cycle, complex environmental and endocrine regulation make spontaneous sexual maturation difficult under artificial breeding conditions, thereby limiting continuous in vivo studies of early oogonial proliferation and oocyte development [1]. AjOSCL therefore provides a controllable platform for investigating the effects of growth factors, environmental cues, and candidate regulatory genes on female germ cell behavior, and represents a useful model for studying the mechanisms underlying early oogenesis in Japanese eel.

### 4.2. Characterization of AjOSCL as an Oogonial Stem Cell-like Line

In fish, in vitro culture systems for SSCs are relatively well established [18]. Long-term SSC lines have also been established in several fish species, including orange-spotted grouper (*E. coioides*) [24], tiger puffer fish (*Takifugu rubripes*) [51], Yellow River carp (*C. carpio*) [20], and Chinese hook (*Snout carp*) [28]. These cells generally exhibit typical germ cell morphology and express multiple germline-associated markers, while their reproductive potential has been further demonstrated by in vitro differentiation or transplantation assays in some species [20,21,28]. In contrast, progress in the long-term culture of female germ cells has been relatively limited. Several ovary-derived cell lines have been established from sterlet (*Acipenser ruthenus*) [52], barramundi (*Lates calcarifer*) [53], and Chinese soft-shelled turtle (*Pelodiscus sinensis*) [54]. However, some of these cultures contain substantial somatic cell components, whereas others have not been comprehensively characterized for germline properties. Consequently, stable long-term cultures predominantly composed of oogonial stem cell-like cells remain scarce, highlighting the need for reliable in vitro models for studying female germ cell biology of fish.

AjOSCL was established from the immature ovary of Japanese eel and showed typical features of undifferentiated female germ cells, including round morphology and colony-like growth. The cells consistently expressed the germline-associated genes *Vasa*, *Nanos1*, *Nanos2*, *Nanos3*, and *Pgr*, as well as the undifferentiated cell-associated genes *Nanog* and *Oct4*. These characteristics are broadly consistent with those reported for germline cell cultures in other fish species [21,37,53]. However, its designation as a stem cell-like line is currently based mainly on in vitro evidence, including morphology, marker expression, and colony-forming ability. Whether AjOSCL can colonize recipient gonads and contribute to donor-derived gamete production still requires functional validation through transplantation assays [11]. Overall, AjOSCL expands the available in vitro models for fish female germ cell research and provides a useful platform for studying oogonial biology and reproductive regulation in Japanese eel.

### 4.3. Transcriptomic Signatures and Pathways Associated with AjOSCL Maintenance

The molecular characteristics of stem cell-like germline populations are determined not only by the expression of germ cell markers but also by the preservation of regulatory networks that support self-renewal, lineage commitment, and developmental competence [55,56]. In the present study, the distinct transcriptional profiles between AjOSCL and AjOTCL provide further evidence that these two cell populations represent different cellular states. Compared with ovarian somatic cell-like populations, AjOSCL maintained a molecular landscape associated with germline identity and developmental plasticity, suggesting that the establishment of this cell line preserved intrinsic features of oogonial stem cells rather than acquiring a somatic-like phenotype during long-term culture.

The maintenance of germline stemness is closely regulated by interactions between cellular intrinsic programs and extracellular niche signals [57,58]. Previous studies have demonstrated that signaling networks involving ECM [59,60], Wnt [61] and TGF-β [48] pathways play conserved roles in germ cell specification, stem cell renewal, and the regulation of differentiation potential across vertebrates [62]. Moreover, the changes in *Wnt3a*, *Wnt5a*, and *Lef1* indicate potential involvement of canonical Wnt signaling in proliferation and maintenance [63]. In addition, the differential expression of *Bmp4*, *Tgfb1*, and *Gdf6a* is particularly relevant given the close association of TGF-β signaling with ovarian development in Japanese eel [64]. The enrichment of these regulatory programs in AjOSCL suggests that the cultured cells retain components of the signaling framework required for sustaining a permissive germline state. This molecular resemblance to the native germline niche may contribute to the maintenance of self-renewal capacity and provide a favorable basis for subsequent differentiation under appropriate induction conditions.

Importantly, the retention of meiosis-associated molecular programs suggests that AjOSCL maintains developmental competence for the transition from mitotic oogonia toward meiotic differentiation. In vertebrate oogenesis, this process requires coordinated regulation of cell cycle progression, maternal mRNA translation, and maturation signaling pathways [65]. In particular, CPEB-mediated translational control plays a central role in regulating maternal transcript activation during oocyte maturation, while cyclin-dependent regulation contributes to meiotic progression through modulation of maturation-promoting factor (MPF) activity [66,67,68]. Thus, the preservation of these meiotic regulatory features indicates that AjOSCL retains a molecular framework compatible with oogenic development. However, transcriptomic signatures alone cannot confirm functional meiotic completion, and further differentiation assays are required to determine whether AjOSCL can undergo meiotic progression and generate mature oocytes in vitro.

### 4.4. Limitations and Application Prospects of AjOSCL

Although AjOSCL was characterized at cytological, molecular, genetic, and transcriptomic levels, the current evidence primarily supports its oogonial stem cell-like properties, while its differentiation and reproductive potential have not yet been functionally demonstrated. Long-term passaging, single-cell and secondary colony formation, karyotype stability, germline marker expression, ALP activity, and transcriptomic pathway enrichment collectively support sustained proliferation, self-renewal capacity, and a germline-associated molecular phenotype. However, the cryopreservation assay confirmed only the recovery and morphological integrity of the cells and did not establish their ability to undergo meiotic or oogenic differentiation. Future studies should therefore combine in vitro differentiation and transplantation-based functional assays. In vitro induction through retinoic acid- or BMP-related signaling could be used to evaluate meiotic and oogenic competence by monitoring morphological changes and the activation of markers such as *Sycp3*, *Dmc1*, *Gdf9*, *Bmp15*, and *zona pellucida* (*zp*) genes. Because conventional two-dimensional culture lacks ovarian somatic cells, extracellular matrix components, and local paracrine signals [68], co-culture with ovarian somatic cells or three-dimensional culture systems may provide a more physiologically relevant microenvironment for differentiation [69]. More stringent functional validation should subsequently involve germ cell transplantation, as the ability to colonize recipient gonads and contribute to gametogenesis is considered the definitive functional criterion for germline stem cell identity [29,31]. Previous studies have shown that cultured or in vitro-expanded fish germline stem cells can retain developmental competence and, after transplantation into germ-cell-deficient recipients, colonize the gonads and generate donor-derived gametes or even viable offspring [14,52,54]. Establishing an appropriate recipient system and tracing donor-derived cells using specific genetic markers will therefore be essential for progressively validating AjOSCL, from in vitro developmental competence to in vivo gonadal colonization and gametogenic function.

Despite these limitations, AjOSCL provides a stable and experimentally tractable platform for studying genes and signaling mechanisms involved in Japanese eel reproductive development, as well as the effects of growth factors, culture conditions, and environmental cues on female germ cells [21,28,37]. Integration with cryopreservation, germ cell transplantation, and gene-editing technologies may further extend its potential applications in germplasm conservation and surrogate reproduction [70,71]. Thus, the major value of AjOSCL lies in providing a renewable in vitro system that can be progressively optimized and functionally validated for investigating female germ cell biology and reproductive regulation in Japanese eel.

## 5. Conclusions

In this study, we established AjOSCL, an oogonial stem cell-like line derived from immature Japanese eel ovaries and capable of long-term stable culture. AjOSCL showed sustained proliferation and colony-forming capacity, while species identification, karyotype analysis, and germline-related and pluripotency-related markers supported its germline characteristics, self-renewal potential, and genetic stability. Transcriptomic profiling confirms that AjOSCL enriched expression of germline and pluripotency markers, activation of key self-renewal pathways (Wnt, ECM-integrin, and TGF-β), and retention of intrinsic oogenic potential through the meiotic regulatory program. Overall, AjOSCL provides a stable and tractable in vitro model for investigating female germ cell maintenance and differentiation in Japanese eel, with potential applications in germplasm conservation and germ cell engineering, and offers a foundation for future reproductive biotechnology.

## Data Availability

The datasets generated and analyzed during the current study are available from the corresponding author upon reasonable request. The authors affirm that the entire creative process of this work, including ideation, literature review, data analysis, drafting, and revision, was conducted without the assistance of any generative artificial intelligence technologies.

## Supplementary Materials

The following supporting information can be downloaded at: https://www.mdpi.com/article/10.3390/biology15212022/s1. Table S1: List of primer sequences for qRT-PCR; Table S2: List of sequence alignment of sample sequencing data with the selected reference genome; Figure S1: Western blot validation of antibody specificity for Vasa, Nanos2, Nanog, Oct4, and Sox2.
