# Supplementary material for: Establishment and Characterization of a Novel Ovarian Germline Cell Line with Oogonial Stem Cell-like Properties from the Japanese Eel (Anguilla japonica)
Source: Biology (Basel). 2026 Nov 6;15(21):2022. doi: 10.3390/biology15212022 (PMC13761601; doi:10.3390/biology15212022)
Supplement: Supplementary file 1 [file biology-15-02022-s001.zip › biology-4600291-supplementary.pdf]

Table S1: List of primer sequences for qRT-PCR

Gene name	Forward primer（5'→3')	Reverse primer（5'→3')	Product Size
Gapdh	GCCAGCCAGAACATCATC	GACACGGAAAGCCATACC	256 bp
Foxl2a	GCAAAACAGCATACGCCACA	ACTCACGTTTCCACTTGCCA	306 bp
Sox3	TACCGACCACGCAGAAAGAC	TGTTCATGCTGGGATGCTGT	263 bp
Amh	CAGAGGAGAGAGCTGACCCT	GGAAGTGGAAAGTCCGGGAG	333 bp
Cyp19a	CTCCAACCAGATGTTCCTC	GCTGCTTCCTTGTGTTCT	249 bp
Nanog	CAGCGTTCTCAGAGGACCAG	GTACCTCTCCGAAACCCAGC	253 bp
Klf5	ATCCCCGAGGCTAAGAAGGT	AAGATGCTGGTGAACTCCGG	251 bp
Lef1	GCCCTGACATGCCCAATTTC	TTGGCCCGCATCTCTTTCAT	328 bp
Vasa	AAACACGGCATCCCCATCAT	GCTCAGTCCCACCTTTCCTC	304 bp
Nanos1	CAGGAACCCAAGATCTGCGT	CTCCCTTAAGTGGCCGTTGT	239 bp
Nanos2	TGGCCATAACGGTCTCTTCG	TGGTGCTGATGGTGCTTAGG	313 bp
Nanos3	CTCGCTGAACACCTCACTGT	TCGTGAAGCAGGCAATCAGT	283 bp
Pgr	TCCTGTCGGTGAAGTTGTCG	ACCCCGTAGTGACATCCAGA	265 bp
COI	ATCCCTCCCAGTCCTAGCTG	GAACAGGGGGAATCAGTGCA	610 bp
Oct4	CGGCGTCTACTATTCGACCC	TCTCTTTGTGTGTACCCGCC	239 bp
Sox2	CTTAACGCTCACAACACGGC	GGATTTTACCACCGAGCCCA	248 bp
Bmp4	TGTACGTGGACTTCAGCGAC	ATGGAGATGGCGCTGAGTTC	326 bp

Table S2: List of sequence alignment of sample sequencing data with the selected reference genome

Sample ID	Clean Reads	Clean Bases	GC (%)	Q30 (%)	Total Reads	Mapped Reads	Uniq Mapped Reads	Multiple Map Reads	Reads Map to '+'	Reads Map to '-'
AJOTCL1	21,307,247	6,263,531,646	49.56	94.88	42,614,494	36,146,256 (84.82%)	35,306,292 (82.85%)	839,964 (1.97%)	18,702,715 (43.89%)	18,692,579 (43.86%)
AJOTCL2	21,252,021	6,224,137,617	50.05	94.87	42,504,042	35,810,250 (84.25%)	34,915,364 (82.15%)	894,886 (2.11%)	18,594,103 (43.75%)	18,592,089 (43.74%)
AJOTCL3	20,437,632	5,992,017,743	50.33	94.78	40,875,264	34,832,152 (85.22%)	34,012,903 (83.21%)	819,249 (2.00%)	18,008,107 (44.06%)	18,001,294 (44.04%)
AJOSCL1	22,022,104	6,381,375,069	50.83	94.82	44,044,208	37,629,848 (85.44%)	36,607,897 (83.12%)	1,021,951 (2.32%)	19,547,861 (44.38%)	19,604,930 (44.51%)
AJOSCL2	20,495,755	5,923,425,857	50.51	94.75	40,991,510	34,326,355 (83.74%)	33,446,846 (81.59%)	879,509 (2.15%)	17,811,767 (43.45%)	17,844,557 (43.53%)
AJOSCL3	20,943,437	6,083,833,977	50.64	94.70	41,886,874	35,748,300 (85.34%)	34,807,982 (83.10%)	940,318 (2.24%)	18,569,928 (44.33%)	18,595,020 (44.39%)

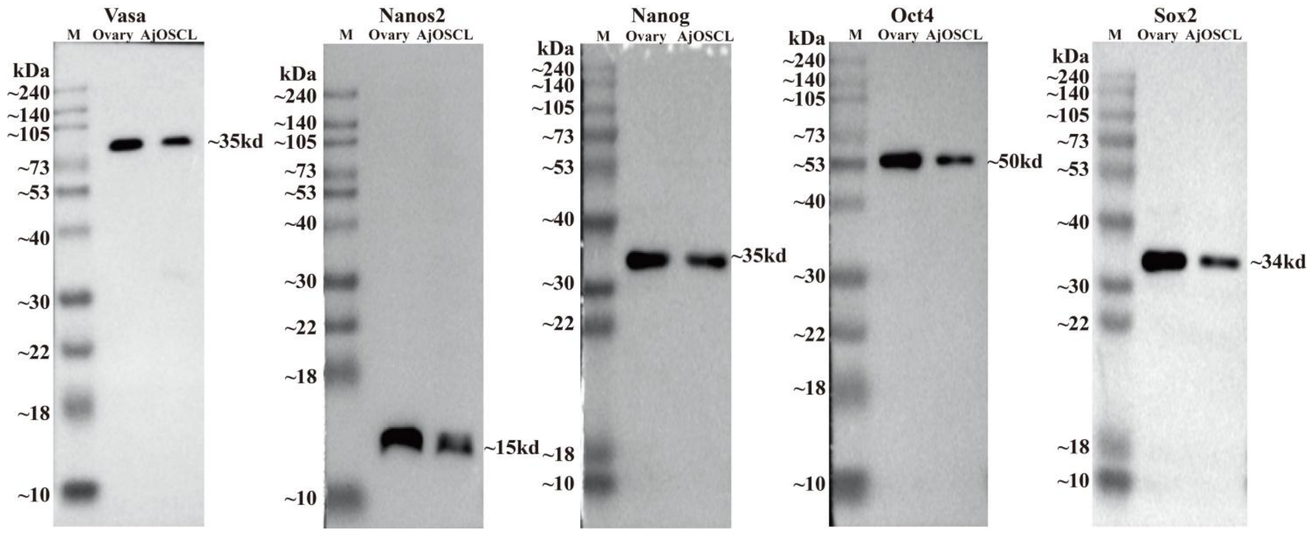

Figure S1: Western blot validation of antibody specificity for Vasa, Nanos2, Nanog, Oct4, and Sox2. Protein extracts from Japanese eel ovary and AjOSCL cells were subjected to Western blot analysis using antibodies against Vasa, Nanos2, Nanog, Oct4, and Sox2. Major immunoreactive bands were detected at approximately 35 kDa for Vasa, 15 kDa for Nanos2, 35 kDa for Nanog, 50 kDa for Oct4, and 34 kDa for Sox2, consistent with the expected molecular weights of the corresponding proteins. M, molecular weight marker.
